# Withdrawal and republication: Interdisciplinary interactive blended learning concept in times of a pandemic – pain medicine “totally digital”

**DOI:** 10.3205/zma001793

**Published:** 2025-12-22

**Authors:** Lisa Schramm, Patrick Friedrich, Jürgen Schüttler, Björn Lütcke

**Affiliations:** 1 University Hospital Erlangen, Department of Anesthesiology, Erlangen, Germany

Schramm L, Friedrich P, Schüttler J, Lütcke B. Interdisciplinary interactive blended learning concept in times of a pandemic – pain medicine “totally digital”. GMS J Med Educ. 2022;39(1):Doc6. DOI: 10.3205/zma001527

## Notification

After publication of the work 

Schramm L, Friedrich P, Schüttler J, Lütcke B. Interdisciplinary interactive blended learning concept in times of a pandemic – pain medicine “totally digital”. GMS J Med Educ. 2022;39(1):Doc6. DOI: 10.3205/zma001527

legally relevant circumstances, particularly in the area of privacy and information protection, have become apparent. 

That is why the article was withdrawn. No fundamental parts of the article are affected, therefore the article has been republished: 

GMS J Med Educ. 2025;42(5):Doc68. DOI: 10.3205/zma001792

The figures affected have been replaced by figures with the same content or by a table with the same content, one figure has been omitted.

## Explanation

The new *figure 2* illustrates the process from the classroom-based concept to blended learning, including the implementation steps; the transition from e-learning to the classroom-based phase is highlighted by an arrow. Elements of the original poster presentation that were not necessary for the article text – including the case vignette, expertise model and bibliography – were deleted and the word cloud was replaced by a summary evaluation. Finally, a brief explanation of the overall process was added to the figure to make the content described in the article text comprehensible at a glance. *Figure 3* was replaced by* table 1* with identical content. *Figure 4* that showed an actor (simulation patient) was not included again due to a withdrawal of consent; this does not affect the content of the article. Since the new figures and the table are identical in content to the original versions, no adjustment of the article text is necessary; the structure of the argumentation and the content presented remain completely unchanged. Due to the omission of one figure and the conversion of another figure into a table, the figure numbering has been adjusted; the former *figure 5* is now *figure 3*, and the former *figure 6* is now *figure 4.*

The team of authors expressly approves the replaced illustrations and the amended publication of the article. The adjustments made relate exclusively to non-essential parts of the article, in particular the consideration of legally relevant aspects of privacy and information protection. The new illustrations and table are identical in content to the previous versions; the text content of the article remains unchanged. 

## Competing interests

The authors declare that they have no competing interests. 

